# Exploring the associations between work addiction, emotional intelligence, psychological detachment, and interpersonal conflicts in young employees

**DOI:** 10.3389/fpubh.2025.1631122

**Published:** 2025-07-09

**Authors:** Jiafan Sheng, Gao Zheng, Huilin Wang, Ziqing Xu

**Affiliations:** ^1^Institute of Science Innovation and Culture, Rajamangala University of Technology Krungthep, Bangkok, Thailand; ^2^School of Business, Hunan University of Science and Technology, Xiangtan, China; ^3^Business School, Guangdong Ocean University, Yangjiang, China

**Keywords:** work addiction, emotional intelligence, psychological detachment, interpersonal conflicts, young employees

## Abstract

**Introduction:**

Work addiction is a growing concern in modern workplaces, particularly among young employees who face intense competition, high performance expectations, and limited work experience. These challenges not only strain their emotional intelligence and psychological detachment but also intensify interpersonal conflicts, negatively impacting workplace dynamics and well-being. This study explores the associations between work addiction, emotional intelligence, psychological detachment, and interpersonal conflicts, providing a deeper understanding of these interconnected factors among young employees.

**Methods:**

This study used a questionnaire survey to collect data from young employees aged 18 to 35 in southern China. The research employed convenience sampling and snowball sampling methods, with 362 valid responses collected. SmartPLS software was used for modeling analysis to verify the hypothesized paths, and bootstrapping with 5,000 samples was applied to test the indirect effects.

**Results:**

The results indicated that all hypotheses were supported. Work addiction was negatively associated with emotional intelligence and psychological detachment, while emotional intelligence was positively associated with psychological detachment. Emotional intelligence and psychological detachment were both negatively associated with interpersonal conflicts. Furthermore, emotional intelligence and psychological detachment were found to mediate the relationship between work addiction and interpersonal conflicts.

**Discussion:**

The findings highlight the need for organizations to address work addiction by promoting a healthier work culture and supporting employees’ emotional and psychological well-being. Encouraging strategies that enhance emotional intelligence and psychological detachment can help mitigate workplace conflicts and improve overall employee relationships.

## 1 Introduction

Interpersonal conflict is a common workplace stressor that triggers cognitive and emotional responses such as anxiety and frustration, negatively impacting employees’ performance and hindering task completion (1). These conflicts are strongly associated with reduced job satisfaction, diminished well-being, and adverse mental health outcomes, including psychological distress, depression, and fatigue, highlighting the urgent need for targeted prevention and management strategies (2). Typically categorized into task and relationship dimensions, interpersonal conflicts can disrupt employees’ psychological empowerment, erode trust, and weaken intentions for knowledge sharing in organizational settings (3). Emotion-driven disputes, in particular, drain organizational resources, provoke negative emotions, and undermine workplace performance, emphasizing the necessity of effective conflict mitigation strategies (4). Conflict management styles, shaped by factors such as hierarchical status differences and organizational culture, play a crucial role in influencing interpersonal dynamics and minimizing the negative effects of conflicts (5). Consequently, effective workplace conflict management is essential, as it determines whether conflicts escalate into detrimental outcomes or transform into opportunities for constructive resolutions, making conflict resolution skills indispensable for roles involving social interactions (6).

For young employees, the challenges posed by interpersonal conflicts are particularly pronounced. On the one hand, young employees are at the early stages of their careers, with relatively limited work experience, making them more susceptible to pressure when dealing with complex interpersonal relationships (7). On the other hand, compared to older employees, young employees are more likely to exhibit emotional reactions, such as anxiety and frustration, when facing workplace conflicts, which may further impact their mental health and work performance (6). Moreover, especially in the current culture of involution, the pressures faced by young employees have intensified (8). This highly competitive environment often pushes young employees to not only strive for exceptional performance but also compete with colleagues for limited resources and career advancement opportunities, making interpersonal conflicts more complex and acute. In such a context, interpersonal conflicts may lead to stronger feelings of frustration and unfairness, posing greater threats to young employees’ mental health and work enthusiasm (9).

Different from previous studies on interpersonal conflicts that primarily focused on their general impact on organizational outcomes (10) or employee well-being (11), this study uniquely examines the associations between young employees’ work addiction, emotional intelligence, psychological detachment, and interpersonal conflicts. Work addiction, characterized by compulsive overwork and an inability to detach, is a growing concern in modern workplaces, particularly among young employees who are in the formative stages of their careers (12). Driven by a desire to prove themselves, achieve rapid career growth, and meet high expectations (13), young employees are especially vulnerable to the pressures of highly competitive environments and the “always-on” culture facilitated by digital technologies (14). This not only strains their mental health and emotional intelligence but also increases the likelihood of interpersonal conflicts and reduces psychological detachment, leading to broader implications for workplace dynamics. By exploring the intricate relationships among these factors, this study provides innovative insights into how work addiction influences interpersonal dynamics and emotional resilience among young employees, addressing a critical gap in the existing literature and highlighting the urgency of targeted interventions.

This study adopts the conservation of resources theory (COR) as its theoretical framework to explore the associations between work addiction, emotional intelligence, psychological detachment, and interpersonal conflicts in young employees. COR theory posits that individuals strive to acquire, maintain, and protect their resources, such as time, energy, and emotional regulation capabilities, to cope with stress (15). Work addiction, characterized by compulsive overwork, often leads to excessive resource depletion, impairing employees’ ability to psychologically detach from work and diminishing their emotional intelligence (16). This resource loss makes young employees more prone to negative emotional reactions, which can escalate interpersonal conflicts (17). COR theory provides a comprehensive lens to examine how resource depletion caused by work addiction triggers a chain reaction that undermines emotional resilience and interpersonal dynamics. By integrating these variables, the framework offers a robust explanation of the complex relationships and highlights the importance of addressing resource management in mitigating the adverse effects of work addiction.

The remainder of this paper is structured as follows: section two focuses on literature review and hypothesis development, introducing the concept of variables and providing a detailed discussion on hypothesis development. Section three outlines the methodology, including participants and procedures, instruments, and data analysis. Section four presents the results, testing the research hypotheses and constructing the structural equation model. Section five provides the discussion, highlighting theoretical contributions and practical implications, while also addressing the study’s limitations. Finally, section six concludes the paper, summarizing the key findings and implications.

## 2 Literature review and hypothesis development

### 2.1 Concept of variables

#### 2.1.1 Work addiction

Work addiction refers to an excessive and compulsive involvement in work (18), where individuals struggle to control their time and energy spent on it, regardless of outcomes (19). This often leads to neglect of personal health, relationships, and social life. It is closely linked to mental health issues like anxiety and depression and can cause physical symptoms like fatigue (20).

Despite growing empirical attention, the construction of work addiction remains contested. Some scholars distinguish it from workaholism, arguing that addiction implies psychological dependency and loss of control, while others use the terms interchangeably (21). Moreover, measurement tools developed in Western contexts (e.g., DUWAS, BWAS) may not fully capture culturally specific expressions of work addiction in collectivist societies like China, where high work involvement can be socially reinforced or even valorized (22). This raises important questions about the cross-cultural validity of existing definitions and the need to contextualize work addiction in terms of normative beliefs about diligence and success.

In the Chinese context, particularly among young employees, several unique cultural and structural factors may exacerbate vulnerability to work addiction. The intense competition in the labor market and internalized social expectations about diligence may contribute to a normative environment that discourages psychological detachment. Additionally, younger workers may lack coping resources or autonomy to set boundaries, making them more susceptible to compulsive work behavior as a maladaptive emotional regulation strategy (23).

While its manifestation varies across cultures, work addiction generally involves spending excessive time and energy on work, making it difficult to stop, and ignoring other life areas (24). Like other addictions, it is hard to self-regulate and often requires external intervention (23). Research indicates that factors such as corporate culture and work stress can worsen the problem (25). These interactions may function as mediating mechanisms, explaining how work addiction leads to emotional exhaustion or interpersonal conflict. A more nuanced understanding of these processes is essential for theory-building and intervention design.

#### 2.1.2 Emotional intelligence

Emotional intelligence is the ability to recognize, understand, manage, and influence emotions in oneself and others (26). It includes skills such as emotional awareness, regulation, empathy, and social interaction, and is essential for decision-making, relationships, and well-being (27). High emotional intelligence is linked to better stress management (28), conflict resolution (29), leadership (30), job performance (31), and mental health (32). The concept draws from psychology, neuroscience, and organizational behavior and is applied across fields like business, education, and healthcare (33). Despite its growing importance, debates continue over its definition, measurement, and potential for development.

#### 2.1.3 Psychological detachment

Psychological detachment refers to the cognitive process of disengaging from work-related thoughts and stressors during non-working hours (34). It is crucial for recovery from work stress, as it allows individuals to mentally disconnect and recharge (35). This detachment has been linked to improved well-being, better sleep, and reduced burnout, as it helps to prevent prolonged work-related rumination (36). In the context of modern work environments, where the boundaries between work and personal life often become blurred, psychological detachment plays a key role in maintaining mental health and overall job satisfaction (37).

#### 2.1.4 Interpersonal conflicts

Interpersonal conflicts refer to disagreements or tensions between individuals due to differences in values, goals, or communication styles (38). These conflicts often arise in both personal and professional settings and can have significant implications for relationships, team dynamics, and overall well-being (39). Unresolved interpersonal conflicts can lead to increased stress, reduced cooperation, and lower job satisfaction (40), while effective conflict resolution strategies are associated with improved collaboration and performance (41). The study of interpersonal conflict is essential for understanding how individuals navigate social interactions and manage disagreements in diverse environments.

### 2.2 Hypothesis development

#### 2.2.1 Work addiction, emotional intelligence, and psychological detachment

Work addiction negatively affects emotional intelligence, as individuals who are overly focused on work often neglect emotional self-regulation (42), impairing their ability to understand and manage emotions (43). This can lead to emotional imbalance and hinder interpersonal relationships (44). Similarly, work addiction is negatively associated with psychological detachment (45). Work-addicted individuals struggle to separate work from personal time, preventing effective psychological recovery and increasing stress (46). The blurred boundaries between work and leisure further hinder recovery and well-being.

In contrast, individuals with higher emotional intelligence manage stress better, regulate emotions effectively, and achieve psychological detachment during non-work hours, promoting mental health. They are also more likely to balance work and personal life, improving overall well-being.

As a result, this study puts forward the following hypotheses:

*Hypothesis 1 (H1)*: There is a negative association between work addiction and emotional intelligence.

*Hypothesis 2 (H2)*: There is a negative association between work addiction and psychological detachment.

*Hypothesis 3 (H3)*: There is a positive association between emotional intelligence and psychological detachment.

#### 2.2.2 Emotional intelligence, psychological detachment, and interpersonal conflicts

Emotional intelligence is negatively associated with interpersonal conflicts. Individuals with higher emotional intelligence tend to better understand and manage their own emotions, possess stronger empathy, and have more effective communication skills (47). These traits enable them to resolve and handle conflicts more efficiently, reducing misunderstandings and easing tense emotions, which ultimately lowers the frequency and intensity of interpersonal conflicts.

Additionally, psychological detachment is negatively associated with interpersonal conflicts (48). Effective psychological detachment allows individuals to recover their energy and emotions during non-work hours, reducing the accumulation of work-related stress (49). When individuals can fully detach psychologically during their off time, they are generally able to maintain a better emotional state and more rational thinking when facing workplace interactions, making them more capable of resolving conflicts (50). In contrast, individuals who lack psychological detachment, due to continuous stress and anxiety, are more prone to conflicts in their daily interactions (35).

As a result, this study puts forward the following hypotheses:

*Hypothesis 4 (H4)*: There is a negative association between emotional intelligence and interpersonal conflicts.

*Hypothesis 5 (H5)*: There is a negative association between psychological detachment and interpersonal conflicts.

#### 2.2.3 Mediation effects

Work addiction indirectly influences conflicts by impairing emotional intelligence and psychological detachment. Individuals with work addiction often neglect emotional regulation, reducing emotional intelligence and increasing the likelihood of misunderstandings and conflicts (51). Additionally, work addiction hinders psychological detachment, leading to accumulated stress and irritability, which heightens interpersonal conflicts (52). In contrast, those who effectively detach from work during off-hours are better able to recover emotionally and reduce conflicts. Thus, emotional intelligence and psychological detachment help explain how work addiction affects interpersonal conflicts (53).

As a result, this study puts forward the following hypothesis:

*Hypothesis 6 (H6)*: Emotional intelligence and psychological detachment mediate the relationship between work addiction and interpersonal conflicts.

**Figure 1:**
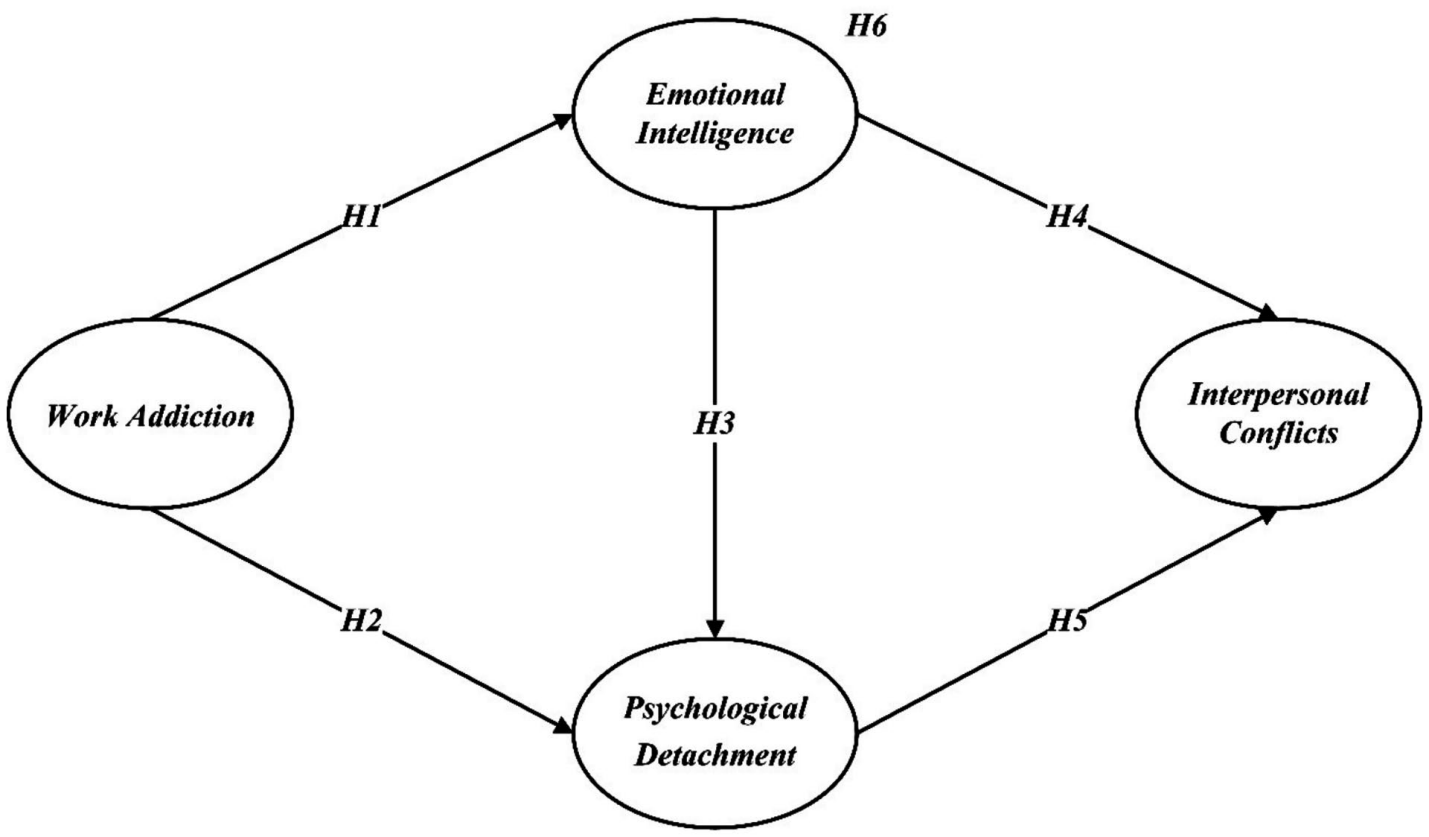
Hypothesized model.

The hypotheses are illustrated in Figure 1.

## 3 Methodology

### 3.1 Participants and procedures

This study conducted an online questionnaire survey among young employees in the workplace. To collect research data, the researchers employed convenience sampling and snowball sampling methods. While these methods facilitated data access, they also limit the generalizability of the findings due to potential sampling bias and lack of randomization. The geographic focus on southern China further restricts the external validity of the results. After obtaining approval from the relevant local authorities, the researchers reached out to young employees aged 18–35 from southern China. The researchers inquired about participants’ interest in the study via the WeChat social media platform and sent the questionnaire link, sincerely inviting them to participate. All participants were informed of the purpose of the survey before completing the questionnaire and voluntarily agreed to participate. Additionally, based on the snowball sampling method, participants who completed the questionnaire were encouraged to invite their friends and teammates to take part in the survey. A total of 405 questionnaires were distributed, with 362 valid responses successfully collected, resulting in an effective response rate of 89.4%.

To minimize the potential threat of common method bias, the questionnaire design included several procedural remedies. These included randomizing item order, ensuring respondent anonymity, and clarifying that there were no right or wrong answers. Nevertheless, common method variance remains a possible limitation and should be addressed in future research through methodological improvements, such as multi-source or time-lagged data collection.

Additionally, given the cross-sectional nature of the data, the study does not claim causal relationships between variables. All interpretations are correlational and should be viewed with caution.

Table 1 presents the demographic characteristics of the 362 young employees participating in this survey. Among the respondents, the demographic characteristics of the young employees participating in the survey are described: (1) 45.3% were male and 54.7% were female; (2) 25.7% of the employees were under the age of 25; (3) 86.5% of the employees held a bachelor’s degree, and 9.9% held a master’s degree or higher; (4) the majority of employees had a monthly income ranging from 5,000 to 12,000 RMB, accounting for 55.6%; (5) 55.8% of the employees slept for 5–7 h per day; (6) 63.8% of the employees frequently engaged in leisure activities, such as sports and entertainment.

**Table 1:** Participants’ demographic characteristics.

Profiles	Category	Frequency	Survey (%)
Gender	Male	164	45.3
	Female	198	54.7
Age	18–25	93	25.7
	26–30	141	39.0
	31–35	128	35.4
Education level	High school or below	4	1.1
	College	9	2.5
	Bachelor’s degree	313	86.5
	Master’s degree or above	36	9.9
Monthly income level	Below 5,000 CNY	80	22.1
	5,000–8,000 CNY	120	33.1
	8,000–12,000 CNY	128	35.4
	Above 12,000 CNY	34	9.4
Average daily sleep duration	Less than 5 h	37	10.2
	5–7 h	202	55.8
	More than 7 h	123	34.0
Do you frequently participate in leisure activities (e.g., sports, entertainment, etc.)?	Yes	231	63.8
	No	131	36.2

Table 2 presents the descriptive statistics for the main variables, including mean (M), standard deviation (SD), and Pearson correlation coefficients (*r*). All variables showed acceptable variability. Work addiction was strongly positively correlated with interpersonal conflict (*r* = 0.667, *p* < 0.01), and moderately negatively correlated with both psychological detachment (*r* = −0.473, *p* < 0.01) and emotional intelligence (*r* = −0.465, *p* < 0.01). These correlations suggest meaningful associations among the variables, consistent with theoretical expectations. However, due to the cross-sectional nature of the data, no causal inferences can be made.

**Table 2:** Descriptive statistics and correlation matrix.

Variable	*M*	SD	1	2	3	4
1. Interpersonal Conflict	2.79	1.20	1.00			
2. Psychological Detach	3.10	1.14	−0.631**	1.00		
3. Emotional Intelligence	3.52	1.06	−0.544**	0.574**	1.00	
4. Work Addiction	3.08	1.12	0.667**	−0.473**	−0.465**	1.00

*N* = 362. **p < 0.01.

### 3.2 Instruments

The questionnaire consisted of five sections. The first section asked respondents to provide demographic information, including gender, age, highest level of education, monthly income level, average daily sleep duration, and whether they regularly participated in leisure activities (e.g., sports, entertainment).

The second section utilized the Bergen Work Addiction Scale (BWAS) developed by Andreassen et al. (21), comprising seven items to collect data on respondents’ work addiction. Sample items included: “Worked so much that it has negatively influenced your health?” The scale was measured using a five-point Likert scale, with response options ranging from 1 (Never) to 5 (Always).

The third section employed the Brief Emotional Intelligence Scale-10 (BEIS-10) developed by Davies et al. (54), consisting of 10 items to gather data on respondents’ emotional intelligence. Sample items included: “I easily recognize my emotions as I experience them.” The scale was measured using a five-point Likert scale, with response options ranging from 1 (Strongly Disagree) to 5 (Strongly Agree).

The fourth section adopted four items from a scale developed by Shimazu et al. (55) to collect data on respondents’ psychological detachment from work. Sample items included: “I do not think about work at all.” The scale was measured using a five-point Likert scale, with response options ranging from 1 (Strongly Disagree) to 5 (Strongly Agree).

The fifth section used the Workplace Interpersonal Conflict Scale (WICS) developed by Carleton et al. (56), consisting of seven items to collect data on respondents’ interpersonal conflict. Sample items included: “Been shown a lack of respect or felt underappreciated by others at work?” The scale was measured using a five-point Likert scale, with response options ranging from 1 (Never) to 5 (Always).

### 3.3 Data analysis

In this research, structural equation modeling (SEM) and SmartPLS software were utilized to analyze the proposed model. SEM is a widely used technique for assessing latent variables in measurement models and testing relationships among latent variables in structural models (57). The two-step modeling method suggested by Anderson and Gerbing (58) was applied, which involves evaluating both measurement and structural models using SEM. In the first step, the researchers assessed the reliability and validity of the measurement tools. The lowest Cronbach’s alpha coefficient for all variables was 0.915, indicating that the instruments exhibited satisfactory reliability and validity. In the second step, the researchers applied the maximum likelihood estimation method to validate the relationships among high-intensity work addiction, emotional intelligence, psychological detachment, and interpersonal conflicts. Additionally, 5,000 bootstrap samples were used to examine the indirect effects between high-intensity emotional intelligence and psychological detachment. Finally, the effectiveness of the model was evaluated, and the fit indices and path coefficients of the hypothesized model were measured.

## 4 Results

### 4.1 Measurement model

The reliability and validity assessment of latent variables incorporated confirmatory factor analysis (CFA) through SmartPLS. All variables demonstrated Cronbach’s *α* values surpassing 0.8 (refer to Table 3), affirming robust internal consistency within the model structure as guided by Fornell and Larcker (59). Additionally, the average variance extraction (AVE) for each variable exceeded 0.6 (as noted in Table 3), surpassing the minimal acceptable threshold of 0.5. Furthermore, the composite reliability (CR) of each latent variable surpassed 0.8, underscoring the model’s robust convergent validity. The resilience of convergent validity across proposed models was well-established. Factor loadings from principal component factor analysis ranged from 0.844 to 0.906 (refer to Table 3), reinforcing the measurement model’s robust construct validity.

**Table 3:** Reliability and validity analysis.

Items	Factor loadings	Cronbach’s α	CR	AVE
Work Addiction (WA)		0.941	0.952	0.740
WA1	0.846			
WA2	0.866			
WA3	0.862			
WA4	0.870			
WA5	0.848			
WA6	0.873			
WA7	0.855			
Emotional Intelligence (EI)		0.963	0.968	0.752
EI1	0.859			
EI2	0.879			
EI3	0.878			
EI4	0.870			
EI5	0.876			
EI6	0.852			
EI7	0.844			
EI8	0.858			
EI9	0.869			
EI10	0.885			
Psychological Detachment (PD)		0.915	0.940	0.797
PD1	0.889			
PD2	0.903			
PD3	0.894			
PD4	0.885			
Interpersonal Conflicts (IC)		0.956	0.964	0.792
IC1	0.889			
IC2	0.897			
IC3	0.890			
IC4	0.880			
IC5	0.880			
IC6	0.906			
IC7	0.888			

Cα, Cronbach’s alpha; AVE, average variance extracted; CR, composite reliability.

Additionally, according to Clark and Watson (60) and Lee et al. (61), when the HTMT value is below the threshold of 0.85, it can be concluded that the discriminant validity of the variables is satisfactory. As shown in Table 4, all correlation coefficients are below 0.85, indicating that all variables exhibit good discriminant validity.

**Table 4:** Discriminant validity test (HTMT).

Construct	EI	IC	PD	WA
EI				
IC	0.565			
PD	0.611	0.674		
WA	0.488	0.702	0.509	

WA, work addiction; EI, emotional intelligence; PD, psychological detachment; IC, interpersonal conflicts.

### 4.2 Structural model

The study evaluated the model fit using the standardized root mean square residual (SRMR). The SRMR value is a standardized residuals index determined by comparing the observed covariance with the hypothesized matrix. A range of SRMR values of 0.1 or lower is considered acceptable (62). Based on the results, the estimated SRMR value was 0.090, which is considered a satisfactory model fit. Additional model fit indices, such as d-ULS and d-G, were within acceptable ranges.

The structural path model in Table 5 and Figure 1 shows that the significant negative correlation between work addiction and emotional intelligence (*β* = −0.467, *t* = 10.703, *p* < 0.001), supporting H1; and there was a significant negative correlation between work addiction and psychological detachment was (*β* = −0.263, *t* = 4.407, *p* < 0.001), supporting H2; and the relationship between emotional intelligence and psychological detachment was statistically significant (*β* = 0.628, *t* = 8.142, *p* < 0.001), supporting H3; and there was a significant negative correlation between emotional intelligence and interpersonal conflicts (*β* = −0,271, *t* = 4.622, *p* < 0.001), supporting H4; and there was a significant negative correlation between psychological detachment and interpersonal conflicts (*β* = −0.476, *t* = 7.228, *p* < 0.001), supporting H5.

**Table 5:** Path coefficients.

No.	Path	*β*	*T* statistics	*p*-values	LLIC	ULIC
H1	WA > EI	−0.467	10.703	0.000	−0.553	−0.384
H2	WA- > PD	−0.263	4.407	0.000	−0.383	−0.149
H3	EI- > PD	0.628	8.142	0.000	0.342	0.557
H4	EI- > IC	−0,271	4.622	0.000	−0.389	−0.162
H5	PD- > IC	−0.476	7.228	0.000	−0.598	−0.337

WA, work addiction; EI, emotional intelligence; PD, psychological detachment; IC, interpersonal conflicts.

**Figure 2:**
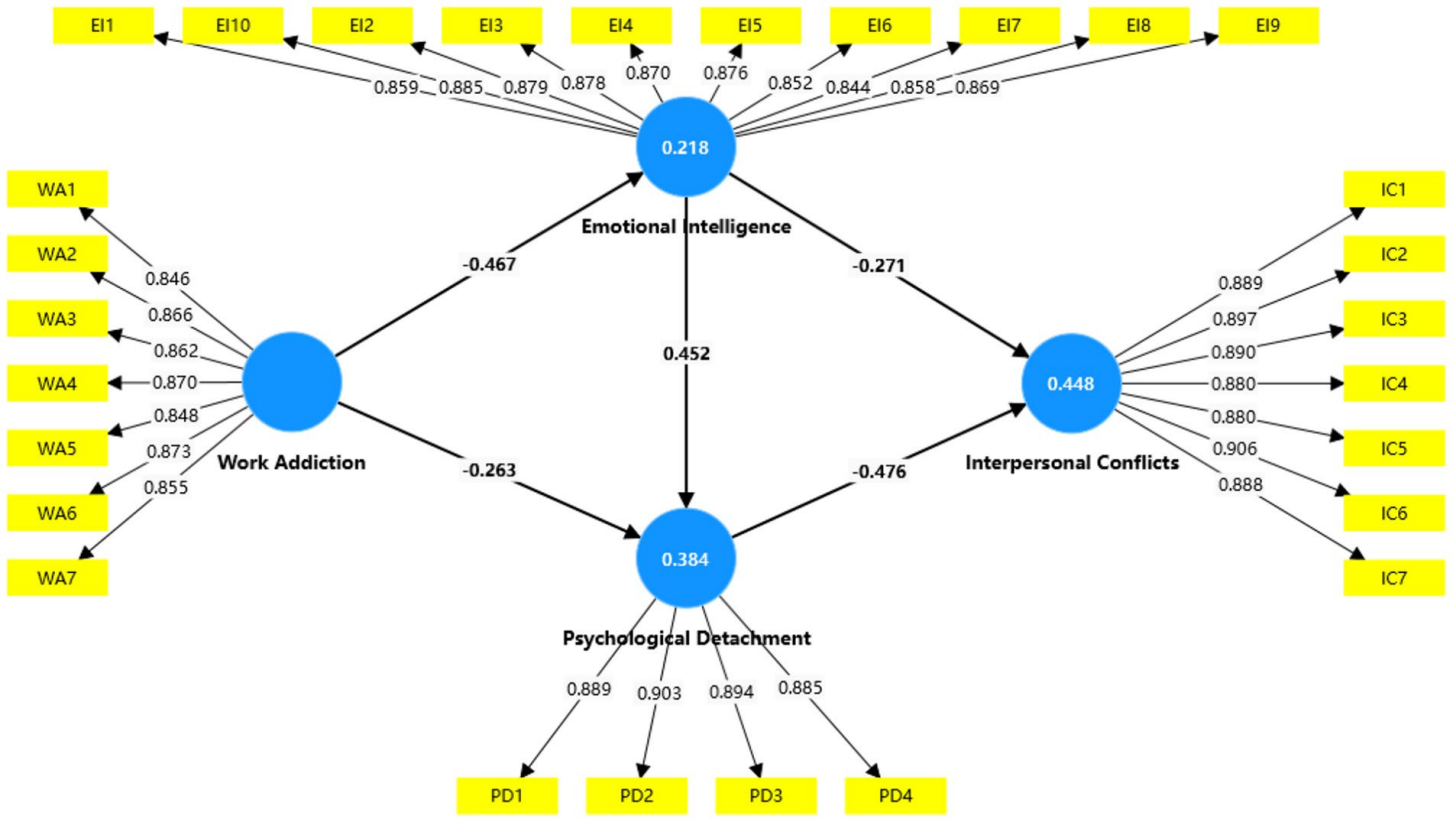
Structural path analysis model.

Model explanatory power was assessed. The goodness of fit of the model is determined by the strength of each structural path determined by the *R*^2^ value for the dependent variable (63), and the value of *R*^2^ should be equal to or greater than 0.1 (64). The results in Table 6 show that all R2 values are greater than 0.1. Hence, the predictive capability is established. Furthermore, Q2 establishes the predictive relevance of the endogenous constructs. A Q^2^ above zero indicates that the model has predictive relevance. The results show that there is significance in the prediction of the constructs (Figure 2 and Table 5).

**Table 6:** Outcomes of *R*^2^ and *Q*^2^ values.

Construct	*R* ^2^	adjusted *R*^2^	*Q* ^2^
EI	0.218	0.216	0.162
IC	0.448	0.445	0.351
PD	0.384	0.381	0.302

EI, emotional intelligence; PD, psychological detachment; IC, interpersonal conflicts.

### 4.3 Mediation analysis

The researchers hypothesized that work addiction influences interpersonal conflicts through two mediators: emotional intelligence and psychological detachment. This study tested the mediation effects using bootstrapping methods. Table 7 shows the emotional intelligence and psychological detachment significantly influenced the relationship between work addiction and interpersonal conflicts (standard indirect effect = 0.352, *p* < 0.001), supporting H6. The results suggest that respondents with lower work addiction who reported higher levels of emotional intelligence and psychological detachment engaged in less interpersonal conflicts.

**Table 7:** Mediation analysis.

No	Path	Original sample (O)	Sample mean (M)	Standard deviation (STDEV)	*T* statistics (|O/STDEV|)	*P*-values
H6	Total indirect effect of WA- > IC	0.352	0.356	0.045	7.84	0.000
	Total Effect of WA- > IC	0.352	0.356	0.045	7.84	0.000

WA, work addiction; IC, interpersonal conflicts.

## 5 Discussion

### 5.1 Theoretical contributions

This study makes several important theoretical contributions to the understanding of work addiction among young employees. First, while there has been considerable research on work addiction, most studies have focused on its impact on the general employee population (18). In contrast, this study uniquely centers on young employees, a group particularly vulnerable to the pressures of high performance expectations, intense competition, and limited work experience (65). These challenges make young employees more susceptible to work addiction, highlighting a critical gap in the literature. By focusing on this group, this study provides a fresh perspective on the specific struggles young employees face in modern work environments. Moreover, it sheds light on how work addiction can trigger a range of emotional and social difficulties within this group, thus offering new insights into the complex relationship between work addiction and interpersonal dynamics among young employees. This novel focus not only contributes to a more nuanced understanding of work addiction but also underscores the need for targeted interventions tailored to the unique challenges young employees encounter in today’s workplace (66).

Importantly, this study extends the application of the COR Theory by illustrating how work addiction leads to the depletion of key psychological resources—namely emotional intelligence and psychological detachment—which are essential for managing workplace stress and interpersonal conflict. According to COR Theory, individuals strive to acquire and preserve resources, and stress occurs when these resources are threatened or lost. Our findings support this theoretical framework by demonstrating that excessive work involvement (i.e., work addiction) accelerates the consumption of emotional and cognitive resources, thereby weakening individuals’ ability to cope effectively with interpersonal tensions.

This study highlights the crucial role of emotional intelligence and psychological detachment in managing interpersonal conflicts in the workplace (67). By examining how work addiction depletes these psychological resources, the research fills a gap in the literature regarding how these factors interact with work addiction and impact conflict resolution (68). These findings further affirm COR Theory’s assertion that resource loss leads to negative outcomes—in this case, conflict escalation—unless individuals can employ alternative coping resources. The findings show that higher emotional intelligence and effective psychological detachment strategies can alleviate interpersonal conflicts and help employees better cope with workplace stress.

Furthermore, the study offers practical guidance for organizational interventions, particularly for young employees facing unique challenges (69). It suggests that organizations design training programs focused on emotional intelligence and psychological detachment techniques to help young employees manage work addiction-related pressures and reduce conflict. These interventions would not only enhance employees’ mental health but also improve their overall performance, thereby fostering better teamwork and a more positive workplace atmosphere.

### 5.2 Practical implications

This study reveals significant associations between work addiction, emotional intelligence, psychological detachment, and interpersonal conflicts, emphasizing the urgent need to address work addiction among young employees. Consistent with the COR Theory, work addiction leads to excessive depletion of emotional and cognitive resources, resulting in stress and interpersonal conflict. Therefore, workplace interventions should aim to conserve and replenish these key resources.

First, creating a work environment that discourages overcommitment is essential. Employers can establish clear boundaries between work and personal life by implementing policies such as limiting overtime, setting reasonable workload expectations, and encouraging employees to take regular breaks. Additionally, promoting flexible working arrangements, such as remote work or adjusted hours, can help employees maintain a healthier balance between their professional and personal lives. A culture that values efficiency over sheer hours worked can reduce excessive engagement with work and foster healthier habits among employees. For example, companies may pilot “email blackout hours” after work, integrate workload monitoring dashboards, or develop leave-taking incentive systems to promote actual disengagement. When implementing such policies, HR managers must assess feasibility in relation to industry demands and resource availability. Potential barriers—such as resistance from senior staff or cultural norms favoring overwork—should be anticipated, and implementation should include leadership endorsement and pilot testing.

Second, fostering emotional intelligence can indirectly help mitigate work addiction by improving employees’ ability to regulate their emotions and manage stress. Organizations can provide emotional intelligence training programs that focus on self-awareness, stress management, and effective interpersonal communication. These programs can equip employees with the skills needed to cope with workplace pressures and reduce reliance on excessive work engagement as a coping mechanism. Additionally, supervisors and team leaders can receive training in emotional intelligence to better support their employees, fostering a more empathetic and understanding work environment that discourages overwork. Organizations may introduce structured emotional intelligence training, delivered through short-format workshops, focusing on specific emotional intelligence competencies such as emotional regulation and empathic listening. Evaluating emotional intelligence development via pre- and post-training assessments can help monitor effectiveness. For cost-conscious organizations, integrating emotional intelligence modules into existing leadership development or onboarding programs may be a more practical approach.

Third, promoting psychological detachment from work is a key strategy to combat work addiction. Employers should encourage employees to engage in leisure activities and hobbies that allow them to disconnect from work-related thoughts. Initiatives such as organizing team-building activities outside of work, providing gym memberships, or offering mindfulness and relaxation programs can support employees in achieving better psychological detachment. Moreover, organizations should advocate for clear communication policies, such as avoiding work-related emails and messages outside of official working hours, to reinforce the boundary between professional and personal time.

Furthermore, interpersonal conflicts often arise from high work demands, stress, and lack of emotional regulation, making it crucial for organizations to address these underlying factors. Implementing conflict resolution training and fostering a culture of collaboration can help employees navigate workplace tensions more effectively. Encouraging open dialog and establishing mechanisms for addressing grievances can prevent conflicts from escalating, ultimately improving workplace relationships and reducing stress levels.

Lastly, organizations should build a supportive workplace culture that prioritizes employee well-being over excessive performance expectations. Managers and leaders play a critical role in setting an example by demonstrating a balanced approach to work and encouraging open discussions about workload and stress management. Providing access to mental health resources, such as counseling or employee assistance programs, can also support employees struggling with work addiction. Additionally, recognizing and rewarding efficiency and productivity rather than long working hours can shift organizational values toward a more sustainable work culture.

By implementing these strategies, organizations can effectively address work addiction among young employees, leading to improved well-being, reduced interpersonal conflicts, and a more sustainable and productive work environment. These measures will not only enhance employee satisfaction and retention but also contribute to long-term organizational success by fostering a healthier and more engaged workforce.

### 5.3 Limitations

The present study has several limitations that should be acknowledged. First, the use of convenience and snowball sampling, along with a geographically limited sample from southern China and a focus on young employees, restricts the generalizability of the findings. Middle-aged or older employees may show different patterns in work addiction, emotional intelligence, psychological detachment, and interpersonal conflict, which were not captured in this study.

Second, the cross-sectional design precludes causal inference. Although associations were observed (e.g., between work addiction and interpersonal conflict), the directionality of these relationships remains unclear. To address this, we have revised causal language throughout the manuscript. Future research should consider longitudinal or experimental designs to establish temporal and causal links.

Third, the reliance on self-reported data raises concerns about common method variance (CMV). Although procedural controls (e.g., anonymity, item randomization) were applied, future studies should adopt multi-source or time-lagged designs and apply statistical remedies to better account for CMV. Additionally, as the examined relationships are already well-established in the literature, future research could explore alternative mechanisms or boundary conditions to enhance theoretical contribution.

## 6 Conclusion

This study explored the associations between work addiction, emotional intelligence, psychological detachment, and interpersonal conflicts among young employees, revealing significant relationships between all variables. To address these associations, employers can support employees in improving psychological detachment through initiatives like flexible schedules, wellness programs, and encouraging disconnection outside work hours. These findings align with COR theory, which holds that individuals strive to protect and build valuable resources. Work addiction may lead to resource depletion, increasing stress and interpersonal conflict. In contrast, emotional intelligence and psychological detachment serve as protective resources that help buffer against these losses. The observed mediation effects reflect typical COR processes, highlighting the importance of reinforcing psychological resources to mitigate the impact of work addiction. Additionally, training programs to enhance emotional intelligence could help reduce interpersonal conflicts and foster a healthier, more collaborative work environment. Future studies should consider longitudinal or experimental designs to clarify causal relationships and reduce method bias. Exploring moderating factors such as organizational support or leadership style could deepen understanding of when these effects are strongest. Cross-cultural research is also needed to assess the generalizability of these findings.

## Data Availability

The raw data supporting the conclusions of this article will be made available by the authors, without undue reservation.
